# Improving medication adherence in adult kidney transplantation (IMAKT): A pilot randomised controlled trial

**DOI:** 10.1038/s41598-019-44002-y

**Published:** 2019-05-22

**Authors:** Jac Kee Low, Elizabeth Manias, Kimberley Crawford, Rowan Walker, William R. Mulley, Nigel D. Toussaint, Michael Dooley, Elaine Kennedy, Catherine L. Smith, Michelle Nalder, Doris Yip, Allison Williams

**Affiliations:** 1 Monash Nursing & Midwifery, Monash University, Clayton Victoria, Australia; 2 School of Nursing and Midwifery, Centre for Quality and Patient Safety Research, Deakin University, Burwood Victoria, Australia; 3 The Royal Melbourne Hospital, Parkville Victoria, Australia; 4 Melbourne School of Health Sciences, The University of Melbourne, Parkville Victoria, Australia; 5 Department of Renal Medicine, Alfred Hospital, Melbourne Victoria, Australia; 6 Department of Medicine, Monash University, Melbourne Victoria, Australia; 7 Department of Nephrology, Monash Medical Centre, Clayton Victoria, Australia; 8 Centre for Inflammatory Diseases, Department of Medicine, Monash University, Clayton Victoria, Australia; 9 Department of Nephrology, The Royal Melbourne Hospital, Parkville Victoria, Australia; 10 Department of Medicine, The University of Melbourne, Parkville Victoria, Australia; 11 Alfred Health, Prahran Victoria, Australia; 12 Centre for Medicine Use and Safety, Monash University, Parkville Victoria, Australia; 13 School of Public Health and Preventive Medicine, Monash University, Melbourne Victoria, Australia; 14 Pharmacy Department, The Royal Melbourne Hospital, Parkville Victoria, Australia

**Keywords:** Patient education, End-stage renal disease

## Abstract

Resources to support long-term medication adherence in kidney transplantation are limited. This study aimed to determine the efficacy of an intervention designed for kidney transplant recipients to enhance medication adherence. A single-blind, multi-site, 12-month pilot randomised controlled trial was conducted at all five public hospitals providing adult kidney transplantation in Victoria, Australia. Participants were recruited at 4 to 6 weeks post-transplantation. Thirty-five participants were randomly assigned to a 3-month intervention, involving a face-to-face meeting (a medication review and a consumer-centred video) and health coaching every two weeks. Thirty-six were randomised to receive usual care. All participants were followed for nine months post-intervention. There were no differences in adherence between groups measured by Medication Event Monitoring System (MEMS), however, it was underutilised by 42% of participants. Based on the self-reported Basel Assessment of Adherence to Immunosuppressive Medications Scale (BAASIS©) score, the percentage of adherent participants decreased significantly between baseline and 3 to 12 months in the control group (*p*-values < 0.001) whilst the percentage of adherent participants in the intervention group remained constant over time. No group differences were detected in other outcomes. Due to the complex medication regimen, developing and testing a medication adherence intervention is difficult in kidney transplantation.

## Introduction

In Australia, kidney transplant recipients (KTRs) are commonly maintained on triple immunosuppressive medication therapy (tacrolimus, mycophenolate and prednisolone) to minimise the risk of rejection and allograft failure^1^. However, non-adherence to immunosuppressive medications is common, with 14% to 36% of KTRs being non-adherent^2–5^. Non-adherence typically emerges during the initial 2 to 12 months following transplantation^6,7^ and the early post-transplantation period (first 3 months) is when the risk of acute rejection is the greatest^8^. Deviation from the immunosuppressive medication regimen will exacerbate the risks associated with inadequate immunosuppression, enhancing the risk of kidney transplant loss. To date, there is very little research identifying optimal approaches to support medication-taking behaviour. A systematic review reported that multidimensional interventions targeting behavioural and emotional aspects have greater potential for improving medication adherence than unidimensional interventions^9^. The aim of this study was to determine whether a 3-month multidimensional intervention was effective in improving medication adherence over 12 months in KTRs.

## Results

### Study participants

**Figure 1:**
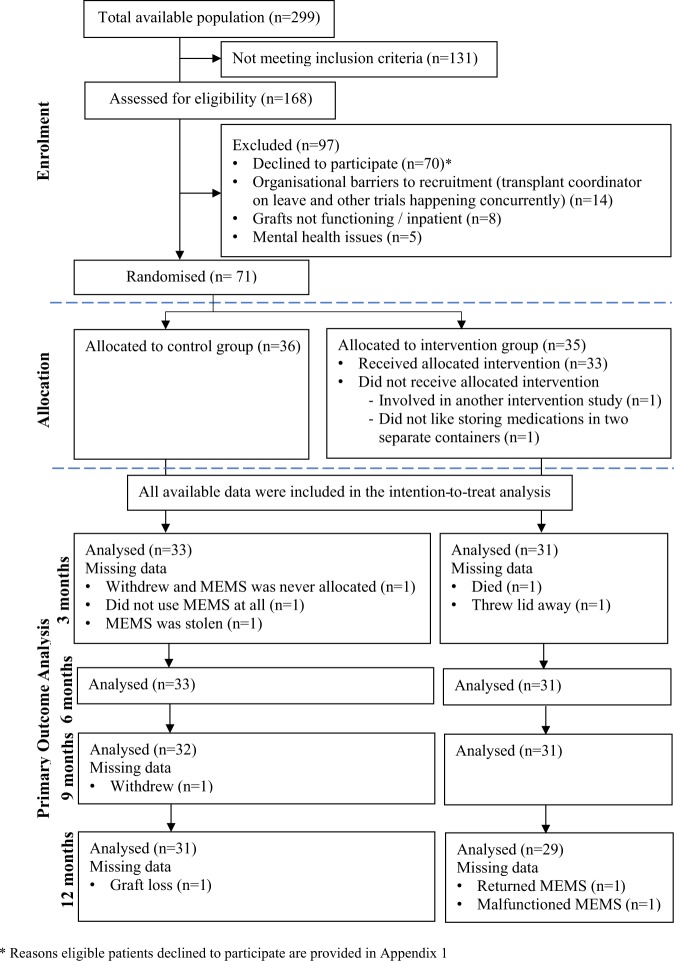
Flow of participants through the 12-month pilot randomised controlled trial.

The flow of participants for the 12-month pilot randomised controlled trial is presented in Fig. 1. The reasons why eligible patients declined to participate are presented in Appendix 1. A total of 36 participants were allocated to the control group and 35 to the intervention group. All participants allocated to the intervention group obtained a one-hour face-to-face meeting and 69% received all 6 telephone calls whilst the remaining participants missed some telephone calls due to work commitment and overseas vacation. The average interval between calls was 16 days, with an average duration of 15 minutes for the first telephone call and 5 minutes for the subsequent telephone calls. Demographics of participants are summarised in Table 1. Compared to those who participated in the trial, the cohort that declined to participate (n = 70) were slightly younger, 44 years old (13.4), and had a higher percentage of male patients (65%).

**Table 1:** Characteristics of participants at baseline*.

Characteristics		Total N = 71	Control n = 36	Intervention n = 35
**Gender**	Male	41 (57.7)	19 (52.8)	22 (62.9)
**Prior dialysis**	Yes	61 (85.9)	33 (91.7)	28 (80.0)
**Type of transplant**	Deceased	50 (70.4)	23 (63.9)	27 (77.1)
**Highest level of education**	Secondary	29 (40.8)	15 (41.7)	14 (40.0)
	Tertiary	42 (59.2)	21 (58.3)	21 (60.0)
**Support at home**	Yes	61 (85.9)	34 (94.4)	27 (77.1)
**Continent of birth**	Australia and Oceania	42 (59.2)	19 (52.8)	23 (5.7)
	Asia	18 (25.4)	13 (36.1)	5 (14.3)
	Europe	8 (11.3)	3 (±8.3)	5 (14.3)
	Africa	3 (4.2)	1 (2.8)	2 (5.7)
**Abbreviated Mental Test Score**	10	53 (74.6)	25 (69.4)	28 (80.0)
	9	15 (21.1)	8 (22.2)	7 (20.0)
	6–8	3 (4.2)	3 (8.4)	0 (0.0)
**Age**	Age years [Mean (SD)]	51.0 (11.4)	48.4 (11.1)	53.6 (11.3)
	Age range	23–74	23–66	32–74
**Time since transplant, days [median (IQR)]**		28.0 (20.3, 41.0)	27.0 (19.3, 39.5)	29.0 (21.0, 43.0)

*Data are numbers (%), means (SD) or median (IQR).

### Primary outcome

Two of the 71 participants did not pick up their Medication Event Monitoring System (MEMS, AARDEX Group, Switzerland) from the hospital pharmacy and withdrew from the study. Of the remaining 69 MEMS that were distributed, 64 devices were returned at the completion of the study including one MEMS that had malfunctioned. The unreturned bottles were due to loss to follow-up (*n* = 2), one reported she discarded the MEMS not realising that she had to return it, one reported he had his bag stolen which had his MEMS inside and another had not used the MEMS at all. The percentage of participants who were not using the MEMS as instructed was higher in male than female participants by 13%, in those who had obtained a kidney transplant from a living donor than from a deceased donor by 19% and in those who had no previous experience of dialysis than those with dialysis experience by 17%. A chi-square test for independence indicated no significant association between MEMS utilisation and adherence score derived from the Basel Assessment of Adherence to Immunosuppressive Medications Scale (BAASIS©), *χ*^2^(1,n = 64) = 0.01, *p* = 0.92, *phi* = 0.05. Further analysis revealed that patients who utilised MEMS adequately were 26.9% more likely to be classified as adherent by the BAASIS inventory, OR = 1.27, 95% CI [0.40, 4.06].

Adherence over 12 months with the MEMS was high for both groups (Intervention: 89.5% (47.7, 98.5); Control: 92.6% (59.5, 96.1)). Three-monthly median adherence rates for each group at each time point are presented in Table 2. The linear mixed-effects model revealed the main effect of time (*F*_(2,61)_ = 0.27, *p* = 0.8) and group (*F*_(1,61)_ = 1.16, *p* = 0.3), and the groupXtime interaction (*F*_(2,61)_ = 0.04, *p* = 0.9) on medication adherence were not significant. Predicted means and SDs (based on the difference in adherence from 3 months) obtained from the linear mixed-effects model together with pairwise comparisons between the groups did not differ between groups.

**Table 2:** Three-monthly median taking adherence (MEMS) by time across groups.

Endpoint	Control			Intervention		
Taking adherence	n (%N)	Median (%)*	IQR	n (%N)	Median (%)*	IQR
**0–12 months**	33 (92)	92.6	59.5, 96.1	31 (89)	89.5	47.7, 98.5
**3 months**	33 (92)	93.5	83.8, 98.4	31 (89)	95.3	87.0, 98.9
**6 months**	33 (92)	94.6	67.4, 98.9	31 (89)	93.4	70.3, 100.0
**9 months**	32 (89)	92.7	38.3, 97.8	31 (89)	91.6	31.0, 98.9
**12 months**	31 (86)	90.2	16.5, 97.6	29 (83)	78.3	13.5, 97.8

*Data are displayed as the median of the percentage of taking adherence. For each individual, quarterly taking adherence was calculated using this formula:

$${\rm{ \% }}\,{\rm{T}}{\rm{a}}{\rm{k}}{\rm{i}}{\rm{n}}{\rm{g}}\,{\rm{A}}{\rm{d}}{\rm{h}}{\rm{e}}{\rm{r}}{\rm{e}}{\rm{n}}{\rm{c}}{\rm{e}}=\,\frac{{\rm{T}}{\rm{h}}{\rm{e}}\,{\rm{n}}{\rm{u}}{\rm{m}}{\rm{b}}{\rm{e}}{\rm{r}}\,{\rm{o}}{\rm{f}}\,{\rm{d}}{\rm{a}}{\rm{y}}{\rm{s}}\,{\rm{w}}{\rm{i}}{\rm{t}}{\rm{h}}\,{\rm{t}}{\rm{h}}{\rm{e}}\,{\rm{c}}{\rm{o}}{\rm{r}}{\rm{r}}{\rm{e}}{\rm{c}}{\rm{t}}\,{\rm{n}}{\rm{u}}{\rm{m}}{\rm{b}}{\rm{e}}{\rm{r}}\,{\rm{o}}{\rm{f}}\,{\rm{M}}{\rm{E}}{\rm{M}}{\rm{S}}\,{\rm{o}}{\rm{p}}{\rm{e}}{\rm{n}}{\rm{i}}{\rm{n}}{\rm{g}}}{{\rm{T}}{\rm{h}}{\rm{e}}\,{\rm{t}}{\rm{o}}{\rm{t}}{\rm{a}}{\rm{l}}\,{\rm{n}}{\rm{u}}{\rm{m}}{\rm{b}}{\rm{e}}{\rm{r}}\,{\rm{o}}{\rm{f}}\,{\rm{m}}{\rm{o}}{\rm{n}}{\rm{i}}{\rm{t}}{\rm{o}}{\rm{r}}{\rm{e}}{\rm{d}}\,{\rm{d}}{\rm{a}}{\rm{y}}{\rm{s}}}\times 100$$.

### Secondary outcomes

**Figure 2:**
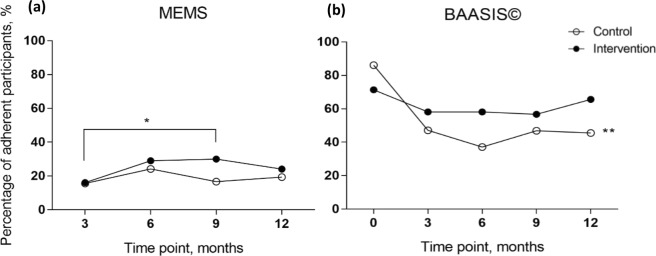
Percentage of adherent participants by time between groups. (**a**) The percentage of adherent participants was defined using the MEMS, as ≥97% of adherence level to both “taking” and “timing” of medication dosing. (**b**) The percentage of adherent participants was defined using the BAASIS© scoring system. *Denotes p < 0.05; **denotes p < 0.001.

The percentage of adherent participants, as measured using the MEMS and BAASIS©, is summarised in Fig. 2. Analyses of the MEMS found no significant associations between the predictor variables (group, time and groupXtime) and the percentage of adherent participants. Pairwise comparisons revealed that more participants were adherent in the intervention group at 9 months than 3 months (*p* < 0.05) whilst no other significant difference was found. The BAASIS© revealed that 71% of the intervention group and 86% of the control group were adherent at baseline. Further analysis conducted using generalized estimated equation revealed no groupXtime interaction effect ${\chi }_{(4)}^{2}$ = 8.7, *p* = 0.068 and group effect ${\chi }_{(1)}^{2}$ = 0.7, *p* = 0.4. However, the main effect of time was significantly associated with the percentage of adherent participants ${\chi }_{(4)}^{2}$ = 21.7, *p* < 0.001. Pairwise comparisons in the control group revealed that the percentage of adherent participants decreased significantly between baseline and 3, 6, 9 or 12 months (*p*-values were < 0.001), with a major change occurring between baseline and 3 months and less notable differences after 3 months. Contrarily, pairwise comparisons within the intervention group revealed the percentage of adherent participants remained stable across all time points.

The 12-month median of timing adherence, derived from MEMS, was 89.5% (46.7, 97.0) for the intervention group and 87.6% (53.7, 94.1) for the control group (Table 3). Median medication possession rates of immunosuppressive medications were similar for both groups: (1) prednisolone and/or hydrocortisone (Intervention: 98.9% (87.8, 100); Control: 94.0% (84.6, 100)), (2) mycophenolate and/or azathioprine (Intervention: 100% (86.5, 100); Control: 100% (100, 100)) and (3) tacrolimus (Intervention: 100% (84.3, 100); Control: 100% (92.0, 100), Table 4). Further analysis on the differences in adherence from 3 months using a linear mixed-effects model revealed a main effect of time on participants’ adherence to the timing of medication-intake (*F*_(2,55)_ = 8.30, *p* < 0.005) and tacrolimus possession rate (*F*_(2,58)_ = 5.2, *p* < 0.05). No other significant main effect of group allocation and time or interaction effects were detected. A Mann-Whitney U test revealed no significant difference in the TacSD between the intervention group, 2.1 (1.8, 2.5), and the control group, 2.3 (1.6, 2.9). Seventy-six percent of the intervention group and 61% of the control group had a TacSD ≤2.5.

**Table 3:** Three-monthly median timing adherence (MEMS) by time across groups.

Endpoint	Control			Intervention		
Taking adherence	n (%N)	Median (%)*	IQR	n (%N)	Median (%)*	IQR
**0–12 months**	33 (92)	87.6	53.7, 94.1	31 (89)	89.5	46.7, 97.0
**3 months**	33 (92)	89.7	72.3, 95.8	31 (89)	91.0	82.1, 96.4
**6 months**	33 (92)	89.3	55.4, 96.7	31 (89)	91.8	66.7, 97.8
**9 months**	32 (89)	84.3	26.9, 96.7	31 (89)	90.1	31.0, 97.7
**12 months**	31 (86)	82.8	39.4, 94.4	29 (83)	77.2	7.4, 97.3

*Data are displayed as the median of the percentage of timing adherence.

**Table 4:** Three-monthly median tacrolimus possession rate by time across groups.

Endpoint	Control			Intervention		
Taking adherence	n (%N)	Median (%)*	IQR	n (%N)	Median (%)*	IQR
**0–12 months**	34 (94)	100	92.0, 100	31 (89)	100	84.3, 100
**3 months**	33 (92)^#^	100	100, 100	29 (83)^#^	100	100, 100
**6 months**	34 (94)	100	99.7, 100	31 (89)	100	100, 100
**9 months**	32 (89)	100	97.8, 100	31 (89)	100	100, 100
**12 months**	32 (89)	100	87.5, 100	31 (89)	100	74.0, 100

*Data are displayed as the median of the percentage of tacrolimus possession. ^#^A lower number of participants at the start of the trial indicates that some participants might have obtained enough supply from their pharmacy prior to the study period and hence, no prescription was dispensed during the first three months.

The Adherence Starts with Knowledge 20 (ASK-20) scores did not differ between groups. A descriptive analysis was conducted at baseline to analyse the percentage of participants who had reported to be having difficulty following the medication regimen, as scored using the ASK-20 survey. At baseline, of the 71 participants, 37% felt that they had too many medications to take in a day, 28% had felt ‘sad, down or blue’ during the month before enrolment and 18% thought that taking medications more than once a day was inconvenient. At 12 months, the top two barriers that were identified at baseline remained unchanged. There remained a large proportion of patients who had felt ‘sad, down or blue’ in the past month (35%) and who believed they had to take too many medications a day (31%). Additionally, 26% reported forgetting things that were important to them from time to time.

## Discussion

Compared to other intervention studies conducted in kidney transplantation aiming to improve medication adherence^3,7,9,10^, our study had the advantage of examining efficacy over 9 months following the 3-month intervention period. Following patients for an additional 9 months was important to check trends in behaviour change post intervention, which other intervention studies have only included 3 months or less. In addition, compared to intervention studies conducted to date^3,7,9,10^, our study utilised a more comprehensive method of measurement by combining objective (electronic monitoring device, MEMS), subjective (self-reported BAASIS© score) and surrogate (prescription refill record and immunosuppressive assay) measures of medication adherence. However, we had a few methodological issues as outlined below, which we believe would be difficult to make this a larger trial unless a better objective measure of medication adherence is available.

The MEMS detected that less than 40% of participants were adherent throughout the study. However, we report that despite being trained and supported, nearly half of the participants did not use the electronic monitoring device as instructed. Thus, determining whether the MEMS findings accurately reflected true adherence level was not possible. The MEMS underutilisation was not accounted for in the sample size calculation. Underutilisation was higher in males, who had a live kidney transplant and had no previous experience of dialysis. This was in accordance with the findings of risk factors related to medication non-adherence^11,12^. However, underutilisation could also be due to research fatigue, resulting from the requirements of daily MEMS monitoring in addition to the complex medication regimen, which involved a medication administration aid for most participants.

During the follow-up period, the MEMS and BAASIS© revealed that approximately one in three individuals were non-adherent by 3 months post-transplantation in both control and intervention groups. However, conflicting results were found, whereby a lower percentage of adherent participants was not associated with a lower MEMS score. This could be due in part to the fact that our study included highly adherent participants, who had an adherence level exceeding 97%, which contributed to the ceiling effect. This was avoided by previous intervention studies by providing the intervention to non-adherent patients after baseline assessment^9^. However, our study was designed to pre-emptively dampen the effects of patients’ perceived barriers and irrational beliefs on compromising medication adherence before non-adherence behaviour was formed. Future studies should consider baseline assessment to firstly, ensure that the device measuring the primary outcome can be implemented successfully in their patient cohort, and secondly, to not include highly adherent patients.

It is perceived that self-reported adherence tends to overestimate adherence in comparison to electronic measures^13^. However, the MEMS approach was coupled with many problems in terms of its execution in this pilot study. It may be specific to the Australian patient group as the majority were given a dose administration aid upon discharge from the hospital and hence, the use of MEMS was inconvenient. In our case, MEMS was less likely to yield reliable data in comparison to the results obtained from BAASIS©. Whilst MEMS was underutilised by 42% of participants, 87% (n = 62) participants had a complete set of BAASIS© data. Although there remained a risk of social desirability bias, self-reported BAASIS© confirmed others’ findings suggesting that medication non-adherence in KTRs occurs early post-transplantation^6^. Given the shortage of kidneys for transplantation and constraints on resources within the healthcare setting, our pilot trial has shown that utilising the BAASIS© to detect immunosuppressive medication non-adherence is an appropriate approach compared with undertaking labour-intensive calculations of medication adherence derived from an electronic monitoring device, prescription refill records and immunosuppressive assays.

One strength of our study was the involvement of participants from all hospitals offering adult kidney transplantation in the state of Victoria, Australia. Victoria is one of two states in Australia with the largest population of KTRs and involvement of patients from all sites within the state increased the applicability of the findings to this population nationally. However, the strength of the study was detracted by methodologic limitations. As there was no centralised system to access all medical records, it remained unknown if the prescription refill record and tacrolimus assays obtained were complete records in those who were obtaining their medications and usual care across multiple providers. It is acknowledged that due to the unexpected underutilisation of the MEMS, the quality of the study was dampened. The lack of effect might be due to the small sample size but could also be due to the intervention not being as effective as we had initially anticipated. Future researchers requiring an electronic measurement of medication adherence may want to also consider ecological momentary assessment using mobile devices, such as smartphones and personal digital assistants. There may be a need to develop an intervention that targets only non-adherent patients or KTRs who speak languages other than English to have a greater impact.

In summary, our study demonstrated that although multiple measures of medication adherence were included, the main obstacle encountered was detecting the effectiveness of the multidimensional intervention using the primary measure. We found the BAASIS© to be an easy-to-use tool, which may be used to detect non-adherence by any healthcare professional trained in its use. The feasibility of measuring medication adherence or intervention efficacy was more difficult than we initially anticipated. A more practical, objective measure of adherence, such as a monitoring device in the form of a dose administration aid, the Maya – Pill Dispenser (MedMinder, United States of America) or SimpleMed + (Vaica, Israel), may provide more accurate results than the monitoring of an index medication.

## Methods

### Barriers

The study was initially designed to be a fully powered randomised controlled trial. However, the trial was underpowered due to the higher than expected number of participants who were not using the MEMS, the device used to measure primary outcome, as instructed. The MEMS is an electronic monitoring device embedded with a microprocessor in the lid of the medication container, which detects the time when the medication bottle is opened. A researcher (KC) demonstrated to participants how to use the MEMS during the one-on-one enrolment face-to-face. All participants could call a helpline for further support anytime during the trial. Further details on the trials and tribulations of MEMS implementation in this study can be found in an article authored by Williams *et al*.^14^.

All participants were asked if they were consistently using their MEMS every 3 months by a researcher (KC). Due to the need to maintain blinding and to minimise the Hawthorne effect, the research team could not follow up participants who were not using the MEMS as requested. During the 3-monthly telephone call, 10 participants admitted to not using the MEMS but in actuality, 27 (42%) of the 64 returned devices were not utilised by the participants as instructed. Non-adherence to MEMS instruction was defined as no daily opening of the MEMS till the end of trial although consistent opening on a daily basis was observed during the initial trial period. However, no participant was excluded based on MEMS underutilisation and all data were included in the intention-to-treat analysis.

### Design

This single-blind, pilot randomised controlled trial was approved by the human research ethics committees of Monash University and all five participating hospitals (HREC/13/SHB/10). All procedures performed in studies involving human participants were in accordance with the ethical standards of the institutional and/or national research committee and with the 1964 Helsinki declaration and its later amendments or comparable ethical standards. Informed consent was obtained from all individual participants included in the study.

All participating sites are public hospitals located in the metropolitan area of Victoria, which altogether conduct approximately 30% of the overall kidney transplant surgeries in Australia^15^. The trial was registered on 3/6/2014 with the Australian New Zealand Clinical Trials Registry (Trial Protocol: ACTRN12614000608662). The datasets generated during and/or analysed during the current study are not publicly available due to ethical considerations but are available from the corresponding author on reasonable request.

Between August 2014 and October 2015, KTRs at 4 to 6 weeks post-transplantation were recruited from the five hospitals that provided acute kidney transplantation in Victoria, Australia. The trial concluded in October 2016 due to funding and time constraints. KTRs were eligible if they were ≥18 years old, comprehended and spoke English, were cognitively intact, and self-managed medications. Patients were excluded if they were visually impaired precluding the ability to view a video or could not answer a telephone call unassisted. Eligible patients who provided verbal consent were contacted by the research team at a routine clinic appointment and were given a Participant Information Sheet/Consent Form during recruitment, upon which written informed consent was obtained.

During enrollment, a researcher (KC) collected participant demographics, cognition level assessed by the Abbreviated Mental Test Score^16^, assessment of medication adherence using the BAASIS^17^ and the ASK-20^18^ which examined barriers to medication adherence. All participants were trained to use the MEMS, the device for measuring the primary outcome, “taking adherence” which was defined as a percentage of days with the correct number of MEMS openings, which could be once or twice daily, over the total number of monitored days.

Participants were randomly allocated to the intervention or control group (1:1) using a group allocation sequence generated by an off-site statistician (CLS), stratified by hospital. Randomisation was conducted by a third party (RSY) blinded to participant identity and the research team was notified via email about the group allocation of each participant. In addition to their usual care, all participants received a telephone call every 3 months from a researcher (KC) blinded to group allocation to collect self-reported data.

Participants in the intervention group received a 3-month intervention (Appendix 2) from a researcher (JKL). Participants in the control group received usual care attending routine outpatient clinic follow-up^19^ and primary care when required. Only two researchers (JKL & AW) were aware of the group allocation.

### Intervention

The 3-month intervention, as summarised in Appendix 2, was informed by a systematic review^9^, 25 in-depth interviews with kidney transplant recipients^20^, 5 focus groups with healthcare professionals, a kidney donor and 2 general practitioners, and the Theory of Planned Behaviour^21^. The intervention consisted of a face-to-face meeting (a medication review and a consumer-centred video)^22^ and a series of 6 fortnightly telephone calls (health coaching).

### Outcomes

A summary of the outcomes and measurement methods is outlined in Table 5. The primary outcome, “taking adherence”, was derived from the percentage of correct number of MEMS openings over the monitoring period (Appendix 3). The MEMS were distributed by either a pharmacist or a kidney transplant coordinator at each site, who was blinded to group allocation, and the same person collected the MEMS data. Among the most commonly prescribed immunosuppressive medications, nephrologists, pharmacists and transplant coordinators at all participating hospitals chose prednisolone as the safest and most practical index medication to use with the MEMS. Compared to tacrolimus, dose reduction of prednisolone was more predictable, making it the preferred choice as an index medication among the health professionals. Secondary outcomes are listed in Table 5.

**Table 5:** The varying measures used to assess medication adherence.

Outcomes	Measures	Prescribed medications(s)	Calculation of adherence level	Data collection
**Taking adherence**	Electronic monitoring device, Medication Event Monitoring System (MEMS)	Prednisolone and/or hydrocortisone	A percentage of days with the correct number of MEMS openings, which could be once or twice daily, over the total number of monitored days	Patients were in charge of when they wanted their MEMS scanned by either a pharmacist or a kidney transplant coordinator
**Percentage of adherent patients**	Electronic monitoring device, Medication Event Monitoring System (MEMS)	Prednisolone and/or hydrocortisone	Patients were categorised as adherent when their “taking adherence” and “timing adherence” were ≥97%	As above. Patients were in charge of when they wanted their MEMS scanned
**Medication adherence and percentage of adherent patients**	Self-reported outcome, Basel Assessment of Adherence to Immunosuppressive Medications Scale (BAASIS©)	Immunosuppressive medications	The original scoring system of the BAASIS© was used to measure adherence without modification and patients were categorised as either adherent (score = 4) or non-adherent (score≥5)	One-off face-to-face during enrolment followed by every 3 months via telephone
**Timing adherence**	Electronic monitoring device, Medication Event Monitoring System (MEMS)	Prednisolone and/or hydrocortisone	A percentage of days on which the MEMS was opened within a 2-hour interval of the patient’s average time, which could be once or twice daily, over the total number of monitored days	Patients were in charge of when they wanted their MEMS scanned
**Medication possession rate**	Prescription refill record	Prednisolone and/or hydrocortisone, mycophenolate and/or azathioprine and tacrolimus	A percentage of the number of doses filled by pharmacy over the number of doses prescribed	At the end of the study, from all community and/or hospital pharmacy(ies)
**Variability of tacrolimus level (TacSD)**	Immunosuppressive assay from hospital medical records	Tacrolimus	Standard deviation was calculated for each participant over the monitoring period.	At the end of the study, from hospital medical records
**Barriers to medication adherence**	Self-reported barriers outcome, Adherence Starts with Knowledge (ASK-20)	All medications	The original scoring system of the ASK-20 was used	One-off face-to-face during enrolment followed by every 3 months via telephone

### Statistical analysis

Statistical analyses were conducted using the SPSS Statistics software (version 22.0, IBM, New York, United States) and were based on intention-to-treat. A two-tailed significance level of 0.05 was used for all statistical analyses. Categorical data are presented as frequencies and percentages. Normally distributed data are presented as means and standard deviations (SD) while non-symmetrical data as medians and inter-quartile range (IQR: Q1, Q3). A main effect of centre was not included in all analyses due to the low number of participants (n = 2) at 2 centres.

A linear mixed-effects model was used to analyze repeated measures of longitudinal medication adherence data, analyzing correlated data resulting from repeated measurements of participants^23^. In order to satisfy model assumptions, a new variable was derived by subtracting the value of 3 months from the value obtained at other time points (6, 9 and 12 months) to calculate the “difference from 3 months”. Consequently, the “difference” was incorporated as the dependent variable for analysis with group, time and a “groupXtime” interaction as the predictor variables, while adjusting for the percentage of adherence at 3 months. The most suitable correlation structure was determined from the data using the Schwarz’s Bayesian Criterion. The F-test for the groupXtime interaction was reported together with pairwise comparisons between the groups at each time point. The same approach was undertaken to analyze timing adherence and medication possession rate. A Mann-Whitney test was conducted to compare the TacSD (intra-patient variability of tacrolimus level) between groups. A generalized estimated equation was performed on the percentage of adherent participants, with the binomial MEMS or BAASIS© score as the dependent variable, with group, time and a groupXtime interaction as the predictor variables. Further analysis using chi-square test was conducted to examine the association between the MEMS utilisation and BAASIS© adherence. A mixed-design analysis of variance was conducted to examine the difference between groups at baseline and 12 months, with the ASK-20 score as the dependent variable.

## Supplementary information

Appendices
