# Supplementary material for: Improving medication adherence in adult kidney transplantation (IMAKT): A pilot randomised controlled trial
Source: Sci Rep. 2019 May 22;9:7734. doi: 10.1038/s41598-019-44002-y (PMC6531445; doi:10.1038/s41598-019-44002-y)
Supplement: Supplementary file 1 — Appendices [file 41598_2019_44002_MOESM1_ESM.pdf]

**Title:** Improving medication adherence in adult kidney transplantation (IMAKT): A pilot randomised controlled trial


Jac Kee Low, BSc (Hons), PhD[1,2*], Elizabeth Manias, RN, PhD, Master of Nursing, MPharm[2,3,4], Kimberley Crawford, PhD[1], Rowan Walker, MD (Thesis), MPH[5,6], William R Mulley, BMed (Hons), PhD[7,8], Nigel D Toussaint, MBBS, PhD[9,10], Michael Dooley, BPharm, Grad Dip Hosp Pharm, PhD[11,12], Elaine Kennedy, RN, BN[7], Catherine L Smith, MPH, MSc(Stats)[13], Michelle Nalder, BPharm (Hons), MClinPharm[14], Doris Yip, Post Grad Dip Acu, Bachelor in Nursing Administration[9], Allison Williams, RN, PhD, Master of Nursing[1]

[1] Monash Nursing & Midwifery, Monash University, Clayton, Victoria, Australia

[2] School of Nursing and Midwifery, Centre for Quality and Patient Safety Research, Deakin University, Burwood, Victoria, Australia

[3] The Royal Melbourne Hospital, Parkville, Victoria, Australia

[4] Melbourne School of Health Sciences, The University of Melbourne, Parkville, Victoria, Australia

[5] Department of Renal Medicine, Alfred Hospital, Melbourne, Victoria, Australia

[6] Department of Medicine, Monash University, Melbourne, Victoria, Australia

[7] Department of Nephrology, Monash Medical Centre, Clayton, Victoria, Australia

[8] Centre for Inflammatory Diseases, Department of Medicine, Monash University, Clayton, Victoria, Australia

[9] Department of Nephrology, The Royal Melbourne Hospital, Parkville, Victoria, Australia

[10] Department of Medicine, The University of Melbourne, Parkville, Victoria, Australia

[11] Alfred Health, Prahran, Victoria, Australia

[12] Centre for Medicine Use and Safety, Monash University, Parkville, Victoria, Australia

[13] School of Public Health and Preventive Medicine, Monash University, Melbourne, Victoria, Australia

[14] Pharmacy Department, The Royal Melbourne Hospital, Parkville, Victoria, Australia

***Corresponding author's contact information:** Jac Kee Low (Address: School of Nursing & Midwifery, Centre for Quality and Patient Safety Research, Deakin University, Burwood Highway, Burwood 3125, Victoria, Australia; phone: +61 (03) 9244 6729; e-mail: jackee.low@gmail.com; ORCID no. 0000-0002-7416-8645)

**Appendix 1: Reasons eligible patients declined to participate**

| Declination reasons | Number of participants (n) |
|---|---|
| Preferred to store medications in one container | 22 |
| Overwhelmed with life post-transplantation | 26 |
| Regional patients | 6 |
| Involved in other studies | 5 |
| Did not need extra help | 4 |
| Language barrier | 4 |
| No reason | 2 |
| Did not like receiving phone calls | 1 |

## Appendix 2: The 3-month IMAKT intervention

**One week after enrolment**

### Face-to-face meeting

To conduct a brief medication review

NHMRC: Medicine review chart Date: ____/____/____ Participant: #____ Total number of medications: ___

| Drug Name | Brand Name / Known as | Reason for use | | | | | |
|---|---|---|---|---|---|---|---|
| | | Prevent rejection | Control blood pressure | Control cholesterol levels | Prevent stomach ulcers | Prevent infection | Others |
| Co-trimoxazole | Bactrim | | | | | | |
| Insulin | Novorapid | | | | | | |
| Insulin | Lantus | | | | | | |
| Mycophenolate | Mycophenolate Sandoz | | | | | | |
| Pantoprazole | Somac | | | | | | |
| Prednisolone | Panafcortelone | | | | | | |
| Tacrolimus | Tacrolimus Sandoz | | | | | | |
| Valganciclovir | Valcyte | | | | | | |

To view an 18-minute video created with consumers

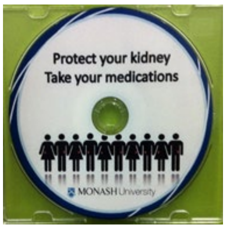


**Two weeks after face-to-face meeting:**
**Health coaching telephone calls every two weeks over a 3-month period**

**First phone call**

Section 1: A general approach was used to ask about participants' identified barriers to medication adherence elicited by the Adherence Starts with Knowledge-20 (ASK-20) tool. However, the ASK-20 was not mentioned during the conversation because it was used to collect data at 12 months as well.

Section 2: To elicit irrational beliefs related to medication intake, guided by the theory of Planned Behaviour.

Section 3: To examine perceived importance and confidence of medication taking using a 10-point Likert scale.

➔ In the event that a barrier was identified during Section 1, 2 or 3, the participant was asked to derive a specific, measurable, agreed upon, realistic, time-sensitive and accountable plan to achieve their goal.

**Appendix 2: The 3-month IMAKT intervention (*continued)***

**An example of when a barrier was identified during Section 2 of the first phone call, when asking the participant about his perceived behavioural control over his medication regimen:**

***Interviewer:*** What do you think makes it difficult for you to take your medications?

***Participant:*** They (the pharmacists) always give us a different brand. The names are too long so we have to read the names on the box but what I did when I went to the chemist, I told them I don't want any generics. I want the original one so we don't confuse them. Sometimes I get three different boxes and three different names (brand names) but the ingredients are exactly the same.

***Interviewer:*** Have you thought about a way around it?

***Participant:*** Well, one I thought… is to mark the boxes 1, 2, 3, 4, 5 and when we receive the box from the chemist, mark them with 1, 2, 3, 4, 5 so I know that I have to take this number (the medication identified by number) in the morning, this number in the afternoon and this number at night.

***Interviewer:*** That is a very clever strategy. Did you come up with that?

***Participant:*** Well, I was an aircraft engineer. That's what we do with the… when we have a lot of parts and some parts are for different devices, so we give them our own number and that number was corresponding to the manufacturing number.

***Interviewer:*** That is a very clever system… that you can use for your medications.

*…The conversation continued with the interviewer encouraging participant to talk more about his derived system and to instil confidence. The participant was subsequently asked when and how the system will be implemented, upon which a specific, measurable, agreed upon, time-sensitive and accountable plan was derived. After concluding the call, the action plan was sent out to the participant in the mail on the same day.*

**My main goal is:** to be able to manage my own medications

**Personal action plan 1:**

I will mark all my boxes of medications when I get the next batch (in two weeks' time). Each drug will have a specific number assigned. For each drug, different strength will be assigned a different letter.

| **Example:** | | | |
|---|---|---|---|
| **Drug Name** | **Brand Name** | **Strength** | **Label** |
| Mycophenolate mofetil | Mycophenolate Sandoz® | 500mg tablets | 1a |
| | | 250mg capsules | 1b |
| Tacrolimus | Tacrolimus Sandoz® | 5mg capsules | 2a |
| | | 1mg capsules | 2b |
| | | 0.5mg capsules | 2c |

Currently, it takes my wife and I 2 – 3 hours to do it. I hope that once we have the new system in place, the amount of time needed will be halved. To show the improvement, I will record the amount of time it takes to put the medications into the 7-day medication box once the new system is in place (_______hours _______minutes).

This change is important because it can be a time-saver and I may be able to arrange the medications for 7 days on my own (when my wife is busy). I am extremely confident about making this change.

**Appendix 2: The 3-month IMAKT intervention (*continued)***

**Second, Third, Fourth, Fifth and Sixth phone call with a 2-week gap in between calls:**

Section 1: To provide a follow up on personal action plan and suggestions provided in previous phone call

Section 2: To enquire if there were any episodes of non-adherence over the last two weeks

➔ Reported ≥1 episode of non-adherence: i) attempt was made to uncover the cause of non-adherence, ii) if the participant displayed no resistance and was willing to make a change, a personal action plan to address non-adherence was derived, and iii) the progress was checked in the subsequent phone call.

> *Note: Whenever the participants mentioned their difficulties, they were encouraged to think of a solution. However, in cases where participants were unable to come up with a solution, the participants were asked if they would like a suggested solution. When a suggestion was provided, the sentence began with "This was an idea of another kidney transplant recipient …" and ended with "What do you think?" to ensure that the participants were empowered; not defeated in the process.*

➔ Reported no episode of non-adherence: i) participants were encouraged to talk about how they managed their medications, and ii) during the phone call, it was reiterated that they were doing a good job and their tips might be used by the interviewer to advice other participants.

## Appendix 3: Medication adherence calculation

A score was calculated to obtain the primary outcome, medication adherence measured using the Medication Event Monitoring System (MEMS). It considers the change in the frequency of medication regimen.

In this example, the patient switches from twice-daily to once-daily regimen of prednisolone 45 days into the trial from the 1st of June and the data is only included up to 92 days to illustrate how medication adherence is calculated. In the study, however, all patients were monitored for up to 365 days.

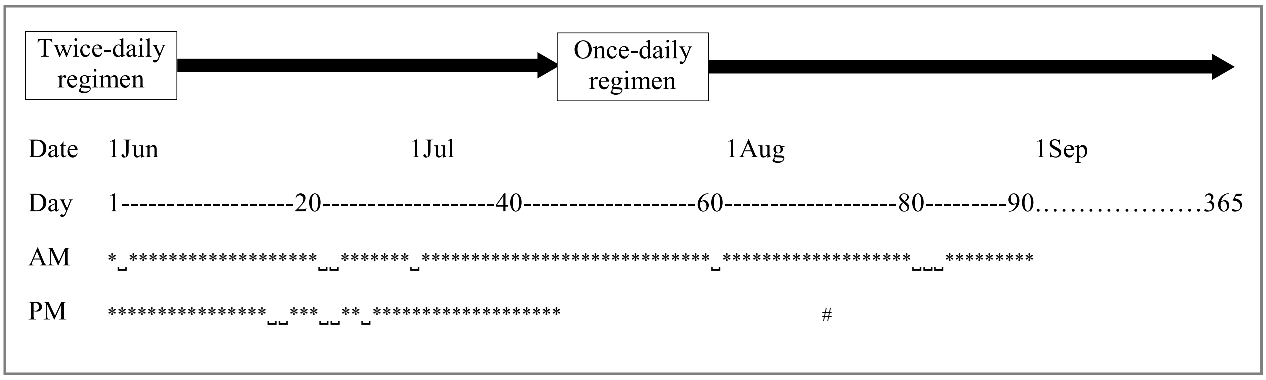


* : dose taken; ␣ : dose not taken; # : dose taken wrongly

First, a score is calculated for each day, with a binomial score of 0 and 1. A score of 0 is given when the frequency of MEMS opening does not correspond with the prescribed dosing regimen.

From 1 June to 1 September, this patient obtained the following scores:

- Daily adherence score = 1 on 80 days
- Daily adherence score = 0 on 12 days

3-month medication adherence is then calculated as: $\frac{(80)}{92}\ x\ 100 = 86.96\%$
